# Declines in Sex Ratio at Birth and Fetal Deaths in Japan, and in U.S. Whites but Not African Americans

**DOI:** 10.1289/ehp.9540

**Published:** 2007-04-09

**Authors:** Devra Lee Davis, Pamela Webster, Hillary Stainthorpe, Janice Chilton, Lovell Jones, Rikuo Doi

**Affiliations:** 1 Center for Environmental Oncology, University of Pittsburgh Cancer Institute and Department of Epidemiology, Graduate School of Public Health, University of Pittsburgh, Pittsburgh, Pennsylvania, USA; 2 Feinberg School of Medicine, Northwestern University, Evanston, Illinois, USA; 3 Center for Research on Minority Health, University of Texas M.D. Anderson Cancer Center, Houston, Texas, USA; 4 Yokohama City University, School of Medicine, Yokohama, Japan

**Keywords:** African Americans, environment, fetal deaths, Japan, race, sex ratio, trends

## Abstract

**Background:**

The expected ratio of male to female births is generally believed to be 1.05, also described as the male proportion of 0.515.

**Objectives:**

We describe trends in sex ratio at birth and in fetal deaths in the United States, in African Americans and in whites, and in Japan, two industrial countries with well-characterized health data infrastructures, and we speculate about possible explanations.

**Methods:**

Public health records from national statistical agencies were assembled to create information on sex ratio at birth and in fetal deaths in the United States (1970–2002) and Japan (1970–1999), using SPSS.

**Results:**

Sex ratio at birth has declined significantly in Japan and in U.S. whites, but not for African Americans, for whom sex ratio remains significantly lower than that of whites. The male proportion of fetal death has increased overall in Japan and in the United States.

**Conclusions:**

Sex ratio declines are equivalent to a shift from male to female births of 135,000 white males in the United States and 127,000 males in Japan. Known and hypothesized risk factors for reduced sex ratio at birth and in fetal deaths cannot account fully for recent trends or racial or national differences. Whether avoidable environmental or other factors—such as widespread exposure to metalloestrogens or other known or suspected endocrine-disrupting materials, changes in parental age, obesity, assisted reproduction, or nutrition—may account for some of these patterns is a matter that merits serious concern.

In modern nations today, the proportion of couples reporting difficulties with reproduction ranges from one of every five to one in four. The survival of any population depends on the capacity of individuals to reproduce successfully if and when they chose to do so. It is increasingly clear that exposures to foreign substances in either parent before conception can have a broad range of teratogenic effects, including reproductive failure, structural or functional defects or altered expression of sex at birth (James 1987, 1998a, 1998b). It is well known that various nutritional, physical, and chemical exposures of pregnant females can affect reproductive success and the health of offspring. Moreover, evidence has mounted that paternal nutrition and a number of behavioral, environmental, and workplace factors affect the DNA of sperm produced (Somers 2004) as well as the tendency to father children with birth defects (Garry et al. 1996; Ryan et al. 2002).

Within any population, the determination of sex at birth is understood to be the result of complex paternal and maternal factors. For any single pregnancy, the *SRY* (sex-determining region Y) gene located on the Y chromosome determines the sex of a fertilized egg. But events occurring after conception may differentially affect the viability of a male conceptus. There is a normalized rate of the proportion of male and female births: Most often, for every 100 female births, 105 male births typically occur (United Nations 2004), which can also be expressed as the male proportion of 0.515. Deviations from this normalized rate and trends in sex ratio overall can provide clues about underlying shifts in population-wide factors that affect the probability that a male conceptus will survive pregnancy. As with many population-wide effects, when it comes to what determines sex ratio at birth, timing of exposure may be more critical than the total dose or rate of exposure (Davis et al. 1998).

To assess recent trends in sex ratio, we expanded work previously published on patterns of recorded sex at birth in Japan and the United States from 1970, including data for white and African-American U.S. subpopulations. These two countries were chosen because they are industrial nations with well-established systems for collecting such data and relatively complete ascertainment of sex at birth. We also examined trends in the sex ratio of fetal deaths in these countries. Given these findings, we explored theoretical explanations for these recent trends in light of studies of highly exposed populations of male workers and propose further studies.

Along with parental age, birth order of child, maternal illness, parental hormone levels, stress, and natural disaster and war, sex ratio has also been shown to vary by racial/ ethnic origin of mother (e.g., Mathews and Hamilton 2005). The numerous studies on the topic present highly varied and sometimes conflicting results (Catalano 2003; Gutierrez-Adan et al. 2000; James 1987; Misao et al. 1998; Parazzini et al. 1998). Marcus et al. (1998) found that patterns of sex ratio differed between U.S. whites and African Americans, with the latter showing a slight increase. They noted that differences in estrogen and testosterone levels between African-American and white women may contribute to differences in the sex ratio of their offspring. A recent study of the Aamjiwnaang First Nation community in Canada reported that sex ratios have dropped from an expected 0.55–0.54 range to 0.45 for the late 1990s, to 0.35 for the 1999–2003 period (Mackenzie et al. 2005). To our knowledge, this is a more significantly reduced sex ratio and greater rate of change than has been reported previously anywhere, and strongly suggests that sex ratio may prove to be an environmentally sensitive indicator.

## Paternal Exposures

Paternal exposures before conception have been found to affect the sex of offspring. Men exposed to dibromochloropropane, certain pesticides, alcohol, lead, and solvents, as well as men employed in the aluminum industry and in saw mills, father fewer sons than expected (Davis et al. 1998). Mocarelli et al. (2000) observed a decreased sex ratio in the offspring of men exposed to dioxin from an industrial accident in Seveso, Italy, in 1976. In the first decade after a chemical plant explosion caused unusually high exposures to dioxin, of the children born to those with the highest exposures, all were female. In another study, Egeland et al. (1994) showed that men who were occupationally exposed to dioxin had altered gonadotrophin and testosterone levels; the researchers hypothesized that altered paternal hormone levels might affect the viability of Y-bearing sperm or the fetuses conceived by them. A follow-up study failed to find significant changes in sex ratio of offspring or fetal death rates in this small sample population (Schnorr et al. 2001). A recent study of men and women working in a Russian plant that manufactured the herbicide 2,4,5-trichlorophenoxyacetic acid found that only 38% of the children fathered by these exposed workers were male, whereas exposed mothers produced a normal 51% males (Ryan et al. 2002). An investigation of the effect of a variety of chemicals on the sex ratio of chemical industry workers in Japan was, in some ways, inconclusive (Okubo et al. 2000): Although researchers found that exposure to chemical materials may have affected the sex ratio of the offspring of the workers, they could not indicate with certainty which chemical materials may have been responsible. The workers’ exposure to heat, they hypothesized, may have been a co-contributor.

## Maternal Exposure

Maternal exposure can also be an important determinant of sex ratio. Maternal serum exposure to polychlorinated biphenyls (PCBs) from the consumption of contaminated Great Lakes fish resulted in decreased sex ratio of offspring (Weisskopf et al. 2003). The odds of having a male child decreased by 46% for every unit increase in the natural log of serum PCB concentration. This observation adds to the growing body of evidence that exposure to certain chemicals can alter sex ratio at birth. Moreover, an increased risk of cryptorchidism among sons of female gardeners has been reported in Denmark, suggesting an association with prenatal exposure to chemicals used in farming and gardening (Weidner et al. 1998). There is some evidence that dose and combinations of exposures can have differing impacts on sex ratio (Axelrod et al. 2001). One small study found that mothers with the highest levels of exposure to PCBs gave birth to proportionally more males than females, whereas those with the lowest exposure did not (Sharpe and Skakkebaeke 1993).

## Materials and Methods

We conducted separate analyses for the United States and Japan. For Japan, we examined sex ratio at births and of fetal deaths from 1949 to 1999. We relied on national statistics provided by the Japanese Vital Statistics Bureau (Japan Statistical Yearbook 2000), which are published yearly, but are not available for the postwar years 1944–1946.

For the United States, we examined trends in sex ratio overall, for African Americans, and for whites, from vital statistics data (Martin et al. 2003) from 1970 to 2002. Last, we calculated the U.S. sex ratio of all fetal deaths, using fetal death data files for 1983–1995 (National Center for Health Statistics 2003). We examined trends in the male proportion of live births, and from these we calculated simple linear regressions. We calculated the male proportion of fetal deaths by dividing the number of male fetal deaths by the sum of male and female fetal deaths. Summing male and female fetal deaths should provide a more accurate estimate of male proportion than using total fetal deaths, because total fetal deaths include fetuses of unknown sex.

Data for the United States were taken from vital statistics published by the National Center for Health Statistics (Martin et al. 2003). Data to examine the U.S. sex ratio of fetal deaths were obtained from the National Center for Health Statistics (2003). For every year from 1983 to 1995 a fetal death data file was released for public use, and from these we obtained figures to calculate the male proportion of fetal deaths. We examined only deaths after 20 weeks gestation because most deaths before that time are of unknown sex. Where sex was unknown, that case was excluded from analysis. Mother’s race was used to calculate proportions by race.

## Results

Our data show distinct and unexplained trends in sex ratio in Japan and in the United States. Since 1970, sex ratio (SR) has declined significantly in U.S. whites and Japanese. But in African Americans, SR has increased modestly over time, while remaining lower than that of whites.

**Figure 1:**
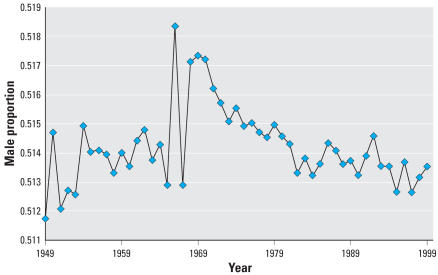
Proportion of male live births, Japan, 1949–1999.

Figure 1 shows the male proportion of live births in Japan from 1949 to 1999. In years before 1970, sex ratio fluctuated greatly from year to year, but since 1970 sex ratio has declined fairly steadily with less yearly fluctuation. For 1970–1999, the regression coefficient is −9.91 × 10^−5^ with an *R* ^2^ of 0.70. Over the study period of 1970–1999, SR for Japan declined significantly (*p* < 0.01) from 0.5172 to 0.5135. This is equivalent to a decline of 37 males per 10,000 births. If the 1970 male proportion had remained constant, this would correspond to a shift from male to female births of approximately 127,000 over these three decades.

**Figure 2:**
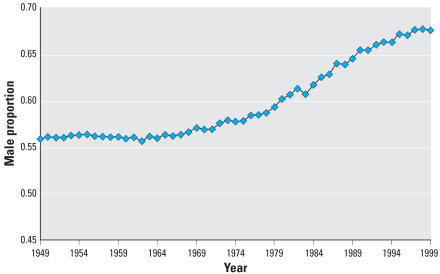
Proportion of male fetal deaths, Japan, 1949–1999.

In Japan, fetal death rates are typically more than twice those of the United States, and range to as much as five times as large. For example, in 1999 Japan reported 31.6 deaths per 1,000, whereas the 1999 U.S. fetal mortality rate for all races was 6.7 per 1,000. Figure 2 shows the male proportion of fetal deaths. For 1972–1999, the rate of increase is 4.27 × 10^−3^, nearly seven times greater than that of the proceeding 72 years. The proportion of fetal deaths that are male has gone from just over half to nearly two-thirds, a figure that held throughout the 1990s. Male fetuses in Japan, then, are clearly at greater risk of dying than are female fetuses. Although fetal death rates have generally fallen, from around 100 per 1,000 in 1960 to 32 per 1,000 in 1999, the proportion of fetal deaths that are male has continued to increase, from 56% of fetal deaths in 1960 to 67.7% in 1999.

**Figure 3:**
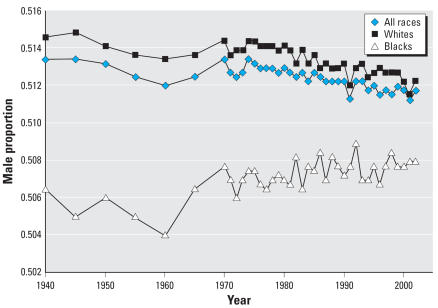
Proportion of male live births, United States, 1940–2002.

Next consider data for the United States, shown in Figure 3. For all races in the United States, the sex ratio decreased from 105.5 in 1970 to a low of 104.6 in 2001. This can be expressed as a decline from 0.5134 in 1970 to 0.5117 in 2002, equivalent to a drop of 17 males per 10,000 births. This trend has a regression coefficient of −0.022 (*p* < 0.001) (or −5.27 × 10^−5^ if expressed as the proportion of births that are male). When U.S. non-Hispanic whites and U.S. African Americans are examined separately, distinct differences are apparent. For U.S. whites, the sex ratio decreased from 105.9 to 105.0 between 1970 and 2002 (and was as low as 104.7 in 2001). This can be expressed as a decline from 0.5143 to 0.5122, equivalent to a drop of 21 males per 10,000 births. This trend for whites has a regression coefficient of −0.029 (*p* < 0.001); that is, the trend for whites was for the sex ratio to drop −0.029 annually since 1970. Assuming that the 1970 sex ratio for all births in the United States had remained constant over these three decades, this is equivalent to a shift from male to female births of approximately 135,000.

For U.S. African Americans, however, the sex ratio increased from 103.1 to 103.2 (expressed as male proportion, 0.5076 to 0.5079), and remained well below that of U.S. whites. This trend has a regression coefficient of 0.013 (*p* < 0.01). In sum, the data show a reduction in the sex ratio for non-Hispanic whites, but no comparable decline for African Americans, whose rates are significantly lower.

**Figure 4:**
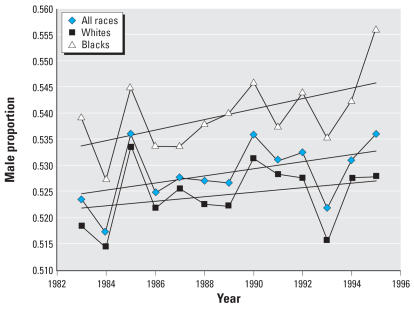
Proportion of male fetal deaths, United States, 1983–1995.

Figure 4 shows the 1983–1995 U.S. trend in the proportion of fetal deaths that are male, separately by race. The male proportion of fetal death has increased most for U.S. African Americans, going from about 0.535 to 0.545 in a decade. The trend for U.S. whites is also slightly upward, with an increase of < 0.005 in the proportion of fetal deaths that are male.

Fetal death rates have declined for all races since 1970, falling roughly in half from 14 to 6.6 per 1,000 in 2000. Although all races have experienced declines in fetal mortality, there are racial disparities in levels of infant mortality. For all years, U.S. African Americans have a higher fetal mortality rate and a higher male proportion of fetal deaths than U.S. whites. In 2000, the fetal mortality rate for African-American mothers was 12.4 per 1,000, compared with a rate for white mothers of 5.6 deaths per 1,000 (Minino et al. 2002).

## Discussion

Previous reports have indicated that in several industrial countries, including the Netherlands, Denmark, Finland (Moller 1996; Vartiainen et al. 1999), Canada (Allan et al. 1997), and the United States, the proportion of males born declined significantly from 1970 to 1990, ranging from an annual drop of 0.001 for the United States (Davis et al. 1998) to 0.003 for the Netherlands (Van Der Palde Bruin et al. 1997). In some Latin American countries (Feitosa and Krieger 1992), the male proportion has fallen by about one male birth per 1,000 live births since the 1970s. Such declining patterns are not evident in some longer term trends. An independent joinpoint analysis by the U.S. Centers for Disease Control and Prevention reported that the trend for whites and Mexicans mirrored the overall downward trend for the last 30 years, but trends for African-American and Chinese mothers rose, and trends for other groups were stable (Mathews and Hamilton 2005) One analysis in Finland (Vartiainen et al. 1999) found that male proportion had increased from 1751 to 1920, and decreased afterward, except for peaks during and after World War I and World War II. Another analysis found parallels between the postwar secular decline of the male:female ratio at birth and the decline of perinatal morbidity and mortality, congenital anomalies, and various constitutional diseases (Jongbloet et al. 2001). Not paternal nor maternal age, nor difference in age of parents, nor birth order can explain these varying time trends (Vartiainen et al. 1999).

The continuing trends in declining sex ratio for U.S. whites and for Japanese that we report here are consistent with those reported recently in other industrial countries, and remain unexplained. Among the explanations suggested for these declines in sex ratio are prenatal exposures to endocrine-disrupting environmental pollutants at a critical stage of sexual differentiation. Researchers theorize that the same chemicals that reduce the sex ratio in the offspring of male workers (Jongbloet et al. 2001) may cause similar effects in populations that are chronically exposed to low levels of these same pollutants.

Not only are fewer males born to some highly exposed cohorts of workers, but surviving male fetuses also appear more susceptible to detrimental effects of toxic exposures than females (Del Rio Gomez et al. 2002; James 1998b; Toppari et al. 1996). Garry et al. (1996) have shown that both reduced SR and higher rates of male birth defects are associated with paternal occupation as a pesticide applicator.

How workplace exposures may contribute to reduced sex ratio in offspring has yet to be explained. Fetal development of male characteristics is a complex process largely determined by the hormonal cascade controlled by the *SRY* gene. Without the proper hormonal cues, even genetically male fetuses will develop along the default female pathway. Sex ratios at birth may be an “important tool” for the investigation of endocrine disruption and a marker of endocrine disruption of one or both parents at the time of conception (Sharpe and Skaakebaeke 1993).

A growing body of evidence indicates that several exogenous factors may be functioning to impair male development. Male reproductive disorders including reduced sperm count and quality, testicular cancer, cryptorchidism (undescended testes), and hypospadias (displacement of the urinary opening toward the scrotum) have been increasing in industrial countries (Del Rio Gomez et al. 2002; Toppari et al. 1996; Weidner et al. 1998). Each of these disorders represents a mild degree of feminization and may have a shared etiologic origin in prenatal exposure to xenoestrogenic endocrine disruptors. Thus, declining sex ratio may be a manifestation of further increased phenotypic feminization of XY fetuses. Evidence that changes in sex ratio could represent a complete phenotypic feminization of genetically male fetuses is provided by observations in animal populations and should be explored further in humans.

Whatever their causes, trends in the sex ratio of fetal death can have important impacts on trends in SR. With respect to the different sex ratio trends in U.S. African Americans and whites, it is important to consider that fetal loss is more common with male fetuses, as indicated by the male dominance in sex ratio of fetal deaths in all populations studied. Improvements in obstetric care for the general population may account for increases in SR that occurred before 1970 in many countries. If fetal deaths in Japan are recorded at 12 weeks and in the United States at 20 weeks, this could account for why the death rates differ so much. But the increasing proportion of males in fetal deaths in the United States and Japan is noteworthy.

In the United States, quality of and access to prenatal obstetric care for African Americans historically has lagged behind those of whites. To the extent that improvements in obstetric care lead to reductions in fetal deaths that occur disproportionately in African-American males, then these advances in care could affect sex ratio at birth. It is possible that improvements in prenatal care may reduce male fetal death rates, with a time lag in the African-American population, as advances have reached this population at a later point in time. The effect of any exposure causing a decline in SR would be more clearly reflected in the white population than the African-American population, because for African Americans recent changes in SR are subject to the confounding effect of drops in the male fetal death rate, as a result of improved prenatal care. As male fetal loss rates decline because of better obstetric care for U.S. African Americans, more male fetuses are expected to survive to birth, leading to an increase in SR. For all years, U.S. African Americans have a lower SR than U.S. whites. Both worldwide and within the United States, sex ratios vary by a few males per 100 females when one examines different racial and ethnic groups. In the United States, this difference could be partly the result of differences in fetal death rates combined with sex ratio of fetal deaths. African Americans have a higher fetal death rate and a higher male proportion of fetal deaths. Combined, these factors could account for reductions in SR for U.S. African Americans compared with U.S. whites by eliminating a larger proportion of male fetuses between conception and birth. Thus, the slight increase in SR for U.S. African Americans since 1970 may be the result of relatively recent advances in prenatal care.

Racial differences in sex ratio in the United States may seem puzzling, though it is possible that what is “normative” varies slightly among groups of different racial heritage. Among the reasons why sex ratio in African Americans could be consistently lower than that of whites are factors that contribute to parental hormonal differences. Some studies have found that greater rates of early onset puberty and premature menarche in young African-American women (e.g., James 2006) whereas others have reported greater exposures to hair care and other personal care products that are contaminated with hormones (e.g., Paulozzi 1999). Whether these factors contribute to a more estrogenic prenatal environment and thereby to a lower proportion of male births, and to greater rates of breast cancer in young African-American women < 35 years of age, are topics that merit serious research.

One other possibility deserves mention. Among African-American young women, obesity is nearly 50% higher than among their white counterparts. Among non-Hispanic white adults 20–74 years of age, 46.8% of women are overweight or obese (body mass index ≥ 25.0). Among non-Hispanic African Americans in the same age group the percentage is 68.3 (Herman-Giddens et al. 1997). In the United States, several observers have speculated that the rise of obesity, while reflecting suburban sprawl, increasing television watching, and inactivity patterns of children, may also be attributed to increased consumption of growth-stimulating and endocrine-disrupting agents in the food supply (Tiwary 1998).

Many developed countries where sex ratio has declined have also seen substantial increases in average body weight and obesity (Centers for Disease Control and Prevention 2002). Moreover, it appears that increased body weight is correlated with a lower sex ratio. At least one study in Africa found that obesity was independently related to a low sex ratio at birth (Andersson and Bergstrom 1998). A declining sex ratio for a population has generally been diagnosed as an indicator of worsening female advantage (Jayaraj and Subramanian 2004). Additional research regarding the role of societal factors in the alteration of sex ratio is needed.

One particular aspect of Japanese culture that may be involved in earlier changes in sex ratio in Japan we do not analyze here. According to one widely held superstition, called *Hinoeuma*, females born in certain years are believed to be stronger than males and will ultimately kill their husbands. Based on this belief, every 60 years when *Hinoeuma* occurs, female babies have been killed by midwives or sometimes even by their family immediately after birth. This is believed to have resulted in a rise in sex ratio in 1906 and again in 1966 (Itoh and Brando 1987).

Regarding changes in SR in Japan, Mizuno (2000) hypothesized that the increase in sex ratio of fetal death in Japan may be affecting the overall rate. Our data suggest that the steady decline in Japanese fetal death rates may be mitigating the impact of Japan’s greatly elevated sex ratio of fetal deaths on SR. Had fetal death rates remained constant, it is likely that SR would have declined even further than it has. Why the sex ratio of fetal deaths in Japan has risen so dramatically since 1970 requires explanation. One possibility we believe merits evaluation is the body burden of mercury or other metalloestrogens, to which male fetuses may be more susceptible. Bioaccumulation of methylmercury can occur in tuna, haddock, and other large fish, an important part of the Japanese diet (Iso et al. 2006). Recent research suggests that a host of prenatal effects occur at intake levels 5–10 times lower than that of adults (Iso et al. 2006). Past research has documented that when severe and widespread methylmercury pollution was experienced in Minamata City, Japan, there was an extreme reduction in the sex ratio at birth of fetal Minamata disease patients, especially in the period of worst pollution and at the worst polluted area (Doi et al. 1987, 2001). The authors also noted that a characteristic seasonal pattern of the birth of male and female fatal Minamata disease patients coincides with the seasonal patterns of the fish catch in Minamata and the outbreak of Minamata disease patients. Lately, Sakamoto et al. (2001) confirmed this independently of Doi et al. (1987, 2001) with the same data, and they also described an increase in male stillborn fetuses.

Strikingly, the severity of birth defects and impairment of sex ratio in Minamata was increased most in those with the highest levels of mercury (Sakamoto et al. 2001).

We hypothesize that the decline in sex ratio in industrial countries may be caused partly by prenatal exposure to metalloestrogens and other endocrine-disrupting chemicals at a critical stage of prenatal development, or paternal exposures that take place before conception that select against expression of the Y chromosome, or some combination of these factors that selectively increase male fetal death rates. Workplace studies and experimental findings support this hypothesis. Recent research in mice offers a fascinating indication that altered gene expression can be affected by chemical factors. Chemically mediated alteration of expression of *Stra8* (stimulated by retinoic acid gene 8) differentially affects the timing of meiosis in the ovary and testes and could influence sex. *Stra8* effects occur during embryogenesis in the female, but occur only after birth in the male (Koubava et al. 2006).

In mammals, meiosis occurs at different time points in males and females, although the mechanism accounting for this is unknown. Although female germ cells begin meiosis during embryogenesis, male embryonic germ cells do not; males undergo G_0_/G_1_ mitotic cell cycle arrest. Only after birth does meiosis begin in the testes. The *Stra8* has been shown to be required for both male and female germ cells to transition into meiosis. Without retinoic acid (RA), an active derivative of vitamin A, meiotic initiation will not occur. RA is sufficient to induce Stra8 expression in embryonic testes and in vitamin A–deficient adult testes *in vivo*. Cytochrome P450 (CYP)-mediated RA metabolism prevents premature Stra8 expression in embryonic testes. Treatment with an inhibitor of RA-metabolizing enzymes shows that a CYP from the 26 family (CYP26) delays Stra8 expression in embryonic testes. Sex-specific regulation of RA signaling thus plays an essential role in meiotic initiation in embryonic ovaries and precludes its occurrence in embryonic testes.

Other explanations may also be relevant. Some studies suggest that psychological stress can lead to a reduction in the sex ratio at birth. A study examining changes associated with a 10-day war in Slovenia documented both a decrease in the sex ratio at birth 6–9 months later, as well as significantly reduced sperm motility among 38 subjects (*p* = 0.01) (Zorn et al. 2002). Similarly, a decline in the sex ratio was documented after the Kobe earthquake in Japan, along with a general reduction in fertility and a lower sex ratio at birth 9 months later (Fukuda et al. 1998). Others have noted a tendency for the sex ratio to decline as socioeconomic development proceeds. The potential roles of delays in first pregnancy, older age of parents, and other factors remain to be further elucidated.

Although the percentage declines in SR reported here are small, because they have occurred in populations of > 400 million, they represent large numbers with considerable public health implications. Declines in the proportion of males born in Japan from 1970 through 1999 and for whites in the United States from 1970 through 2002 are equivalent to a shift from male to female births of 127,000 and 135,000 births, respectively. Fetal death rates are five times higher in Japan than in whites in the United States. Disturbances in SR may provide a sentinel indicator of larger ecologic patterns that can only be corroborated by detailed studies of smaller, more defined cohorts, with specified exposures and well-characterized information on socioeconomic, cultural, and racial factors.

While noting that sex ratio has declined in many regions, one of the foremost authorities on the topic questions whether environmentally prevalent endocrine disrupters could ever be demonstrated to account for such broad patterns (James 1998b). He adds that it is not possible to determine what sex ratios would have been doing absent current pollution patterns or current increasing trends in age of parents. We agree. Sex ratio in a population is the result of multiple determinants that can be identified only through detailed studies of selected subpopulations with unusual exposures or conditions. For example, the health survey underway in Sarnia, Ontario, Canada, to further investigate the pronounced decrease in sex ratio in a First Nation community may shed light on this topic (Mackenzie et al. 2005). In addition, in the data examined here, the possibility that there could be systematic differences in the ascertainment of sex at death cannot be ruled out. If this proves to be the case, it could account for the observed differences in male fetal deaths rates in the United States and Japan. As it stands, the falling sex ratio coupled with the disproportionately male fetal deaths supports the hypothesis that males are being culled in some systematic fashion. In addition, the fact that boys are disproportionately represented in miscarriages (< 20 weeks), indicates that the trends here are underestimates of the true patterns of fetal deaths, as we have restricted our analysis to those that occur after 20 weeks.

Although population-wide trends in sex ratio cannot be attributed to any single set of risk factors, given the importance of reproduction for the health of any species, the continuing trends in reduced births of males in U.S. whites and Japan reported here are matters that merit concern. Efforts should proceed to examine sex ratio in smaller groups with defined exposures as a potential indicator of environmental contamination, and to develop geographic and temporal analyses of patterns in sex ratio as a means of generating hypotheses about avoidable or controllable factors that may account for the trends reported here.

## References

[b1-ehp0115-000941] Allan BB, Brant R, Seidel JE, Jarrell JF (1997). Declining sex ratios in Canada. CMAJ.

[b2-ehp0115-000941] Andersson R, Bergstrom S (1998). Is maternal malnutrition associated with a low sex ratio at birth?. Hum Biol.

[b3-ehp0115-000941] Catalano RA (2003). Sex ratios in the two Germanies: a test of the economic stress hypothesis. Hum Reprod.

[b4-ehp0115-000941] Centers for Disease Control and Prevention 2002. Prevalence of Overweight and Obesity among Adults: United States, 1999–2002. Available: http://www.cdc.gov/nchs/products/pubs/pubd/hestats/obese/obse99.htm [accessed 4 May 2007].

[b5-ehp0115-000941] Davis DL, Axelrod D, Jones LA, Hajek RA (2001). It’s time to rethink dose: the case for combining cancer and birth and developmental defects [Editorial]. Environ Health Perspect.

[b6-ehp0115-000941] Davis DL, Gottlieb MB, Stampnitzky JR (1998). Reduced ratio of male to female births in several industrial countries. JAMA.

[b7-ehp0115-000941] Del Rio Gomez I, Marshall T, Tsai P, Shao Y-S, Guo YL (2002). Number of boys born to men exposed to polychlorinated biphenyls. Lancet.

[b8-ehp0115-000941] Doi R Ohno H Davis DL Harada M 2001. Altered sex ratio at birth in fetal Minamata disease patients [Abstract]. 6th International Conference on Mercury as a Global Pollutant, Minamata, Japan, 15–19 October 2001, 227.

[b9-ehp0115-000941] Doi R, Ohno H, Yamashita K, Harada M (1987). Seasonal changes in the birth of fetal Minamata disease patients. Progr Biometeorol.

[b10-ehp0115-000941] Egeland GM, Sweeney MH, Fingerhut MA, Wille KK, Schnorr TM, Halperin WE (1994). Total serum testosterone and gonadotropins in workers exposed to dioxin. Am J Epidemiol.

[b11-ehp0115-000941] Feitosa MF, Krieger H (1992). Demography of the human sex ratio in some Latin American countries, 1967–1986. Hum Biol.

[b12-ehp0115-000941] Fukuda M, Fukuda K, Shimizu T, Moller H (1998). Decline in sex ratio at birth after the Kobe earthquake. Hum Reprod.

[b13-ehp0115-000941] Garry VF, Schreinemachers D, Harkins ME, Griffith J (1996). Pesticide appliers, biocides, and birth defects in rural Minnesota. Environ Health Perspect.

[b14-ehp0115-000941] Gutierrez-Adan A, Pintado B, de la Fuente J (2000). Demographic and behavioral determinants of male-to-female birth ratio in Spain from 1981 to 1997. Hum Biol.

[b15-ehp0115-000941] Herman-Giddens ME, Slora EJ, Wasserman RC, Bourdony CJ, Bhapkar MV, Koch GG (1997). Secondary sexual characteristics and menses in young girls seen in office practice: a study from the pediatric research in office settings network. Pediatrics.

[b16-ehp0115-000941] Iso H, Kobayashi M, Ishihara J, Sasaki S, Okada K, Kita Y (2006). Intake of fish and n3 fatty acids and risk of coronary heart disease among Japanese: the Japan public health center-based (JPHC) study cohort I. Circulation.

[b17-ehp0115-000941] Itoh T, Brando R (1987). Fertility change of the year of “Hinoeuma.”. Jinko Mondai Kenkyu.

[b18-ehp0115-000941] James WH (1987). The human sex ratio, part 1: a review of the literature. Hum Biol.

[b19-ehp0115-000941] James WH (1998a). Hypothesis on mammalian sex ratio at birth. J Theor Biol.

[b20-ehp0115-000941] James WH (1998b). Re: The use of offspring sex ratios in the search for endocrine disruptors [Letter]. Environ Health Perspect.

[b21-ehp0115-000941] James WH (2006). Offspring sex ratios at birth as markers of paternal endocrine disruption. Environ Res.

[b22-ehp0115-000941] Japan Statistical Yearbook 2000. Japan Statistical Yearbook. Tokyo:Japanese Statistics Bureau, Ministry of Internal Affairs and Communications.

[b23-ehp0115-000941] Jayaraj D, Subramanian S (2004). Women’s wellbeing and the sex ratio at birth: some suggestive evidence from India. J Dev Stud.

[b24-ehp0115-000941] Jongbloet PH, Zielhuis GA, Groenewoud HM, Pasker-De Jong PC (2001). The secular trends in male:female ratio at birth in postwar industrialized countries. Environ Health Perspect.

[b25-ehp0115-000941] Koubava J, Menke DB, Zhou Q, Capel B, Griswold MD, Page DC (2006). Retinoic acid regulates sex-specific timing of meiotic initiation in mice. Proc Natl Acad Sci USA.

[b26-ehp0115-000941] Mackenzie CA, Lockridge A, Keith M (2005). Declining sex ratio in a First Nation community. Environ Health Perspect.

[b27-ehp0115-000941] Marcus M, Kiely J, Xu F, McGeehin M, Jackson R, Sinks T (1998). Changing sex ratio in the United States 1969–1995. Fertil Steril.

[b28-ehp0115-000941] Martin JA, Hamilton BE, Sutton PD, Ventura MA, Menacker F, Munson MS (2003). Births: final data for 2002. Natl Vital Stat Rep.

[b29-ehp0115-000941] Mathews TJ, Hamilton BE (2005). Trend analysis of the sex ratio at birth in the United States. Natl Vital Stat Rep.

[b30-ehp0115-000941] Minino AM, Arias E, Kochanek KD, Murphy SL, Smith BL (2002). Deaths: final data for 2000. Natl Vital Stat Rep.

[b31-ehp0115-000941] Misao F, Kyomi F, Shimizu T, Moller H (1998). Decline in sex ratio at birth after Kobe earthquake. Hum Reprod.

[b32-ehp0115-000941] Mizuno R (2000). The male/female ratio of fetal deaths and births in Japan. Lancet.

[b33-ehp0115-000941] Mocarelli P, Gerthoux PM, Ferrari E, Patterson DG, Kieszak SM, Brambilla P (2000). Paternal concentrations of dioxin and sex ratio of offspring. Lancet.

[b34-ehp0115-000941] Moller H (1996). Change in male:female ratio among newborn infants in Denmark. Lancet.

[b35-ehp0115-000941] National Center for Health Statistics 2003. Fetal Death Data CD-ROM, 1983–1995. Springfield, VA:National Technical Information Service.

[b36-ehp0115-000941] Okubo Y, Suwazono Y, Kobayashi E, Nogawa K (2000). Altered sex ratio of offspring in chemical industry workers. J Occup Health.

[b37-ehp0115-000941] Parazzini F, La Vecchia C, Levi F, Franceschi S (1998). Trends in male:female ratio among newborn infants in 29 countries from five continents. Hum Reprod.

[b38-ehp0115-000941] Paulozzi LJ (1999). International trends in rates of hypospadias and cryptorchidism. Environ Health Perspect.

[b39-ehp0115-000941] Ryan JJ, Amirova Z, Carrier G (2002). Sex ratios of children of Russian pesticide producers exposed to dioxin. Environ Health Perspect.

[b40-ehp0115-000941] Sakamoto M, Nakano A, Akagi H (2001). Declining Minamata male birth ratio associated with increased male fetal death due to heavy methylmercury pollution. Environ Res.

[b41-ehp0115-000941] Schnorr TM, Lawson CC, Whelan EA, Dankovic DA, Deddens JA, Piacitelli LA (2001). Spontaneous abortion, sex ratio, and paternal occupational exposure to 2,3,7,8-tetrachlorodibenzo-*p*-dioxin. Environ Health Perspect.

[b42-ehp0115-000941] Sharpe RM, Skakkebaeke NE (1993). Are oestrogens involved in falling sperm counts and disorders of the male reproductive tract?. Lancet.

[b43-ehp0115-000941] Somers CM, McCarry BE, Malek F, Quinn J (2004). Air pollution lowers the risk of heritable mutations in mice. Science.

[b44-ehp0115-000941] Tiwary CM (1998). Premature sexual development in children following the use of estrogen- or placenta-containing hair products. Clin Pediatr (Phila).

[b45-ehp0115-000941] Toppari J, Larsen JC, Christiansen P, Giwercman A, Grandjean P, Guillette LJ (1996). Male reproductive health and environmental xenoestrogens. Environ Health Perspect.

[b46-ehp0115-000941] United Nations 2004. Live births by age of mother, sex of the child and urban/rural residence: latest available year, 1995–2004. Table 10. In: Demographic Yearbook. Available: http://unstats.un.org/unsd/demographic/products/dyb/DYB2004/Table10.pdf [accessed 27 February 2007].

[b47-ehp0115-000941] Van Der Pal-de Bruin KM, Verloove-Vanhorick SP, Roeleveld N (1997). Change in male:female ratio among newborn babies in Netherlands. Lancet.

[b48-ehp0115-000941] Vartiainen T, Kartovaara L, Tuomisto J (1999). Environmental chemicals and changes in sex ratio: analysis over 250 years in Finland. Environ Health Perspect.

[b49-ehp0115-000941] Weidner IS, Moller H, Jensen TK, Skakkebaek NE (1998). Cryptorchidism and hypospadias in sons of gardeners and farmers. Environ Health Perspect.

[b50-ehp0115-000941] Weisskopf MG, Anderson HA, Hanrahan LP, the Great Lakes Consortium (2003). Decreased sex ratio following maternal exposure to polychlorinated biphenyls from contaminated Great Lakes sport-caught fish: a retrospective cohort study. Environ Health.

[b51-ehp0115-000941] Zorn B, Sucur V, Stare J, Meden-Vrtovec H (2002). Decline in sex ratio at birth after 10-day war in Slovenia: brief communication. Hum Reprod.

